# Supplementary material for: Bet-hedging across generations can affect the evolution of variance-sensitive strategies within generations
Source: Proc Biol Sci. 2019 Nov 27;286(1916):20192070. doi: 10.1098/rspb.2019.2070 (PMC6939271; doi:10.1098/rspb.2019.2070)
Supplement: Supplementary Material [file rspb20192070supp1.docx]

Electronic Supplementary Material (ESM)

to

# Bet-hedging across generations can affect the evolution of variance-sensitive strategies within generations

Haaland, Thomas R., Jonathan Wright, Irja I. Ratikainen

## Supplementary figures


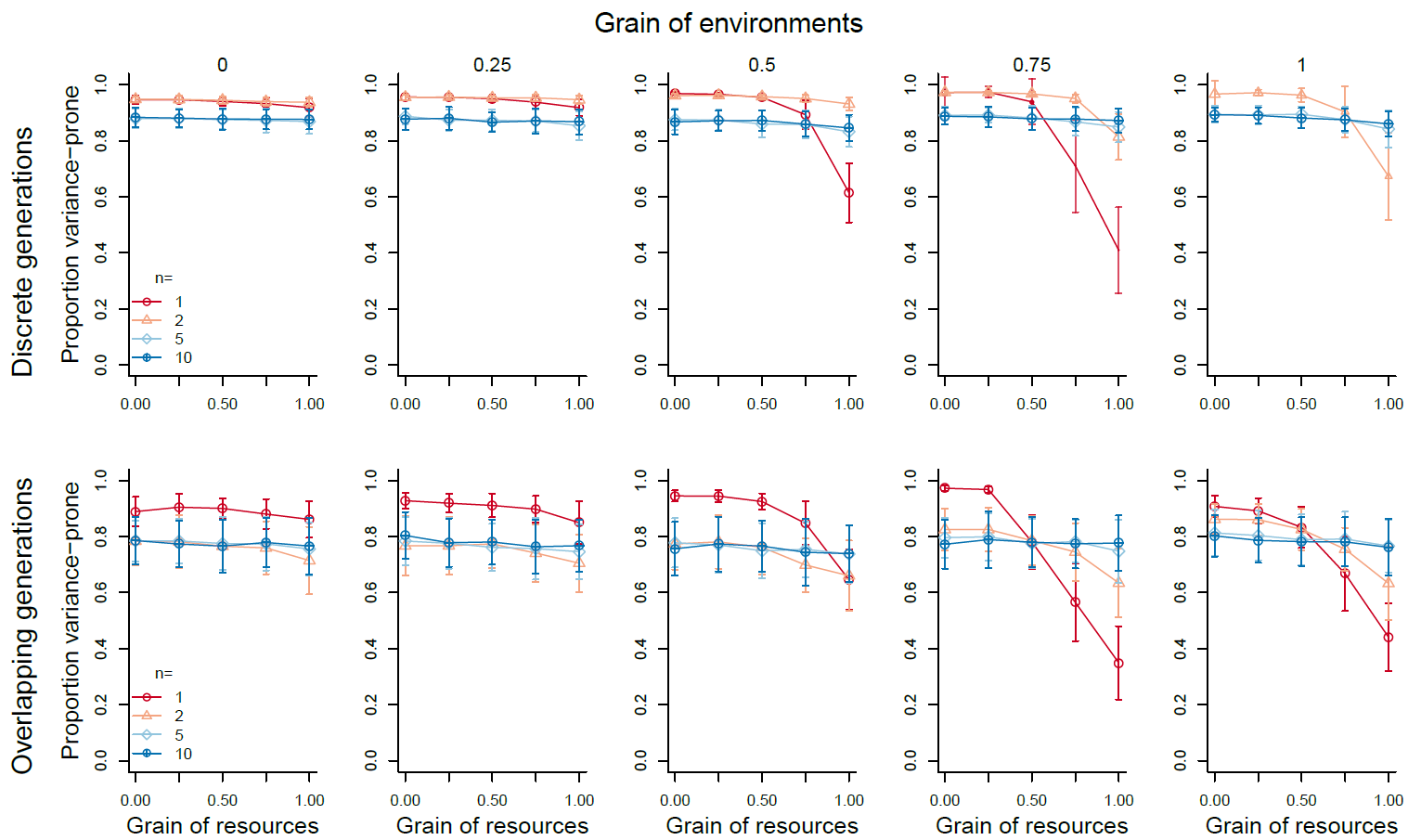


**Figure S1:** Mean evolved gene values for the proportion of the population playing variance-prone strategies at the end of the simulations for discrete (top row, between-year mortality *α*=1) and overlapping (right, *α*=0.5) generations, for different grain of environment (*g_e_*, x-axes), grain of resources (*g_r_*, given by the number 0 to 1 above each column) and number of decision events prior to reproduction (colours, point types). Points indicate means and error bars indicate standard deviations across 100 replicate populations, and relative point size represents the proportion of populations surviving until the end of the simulation.


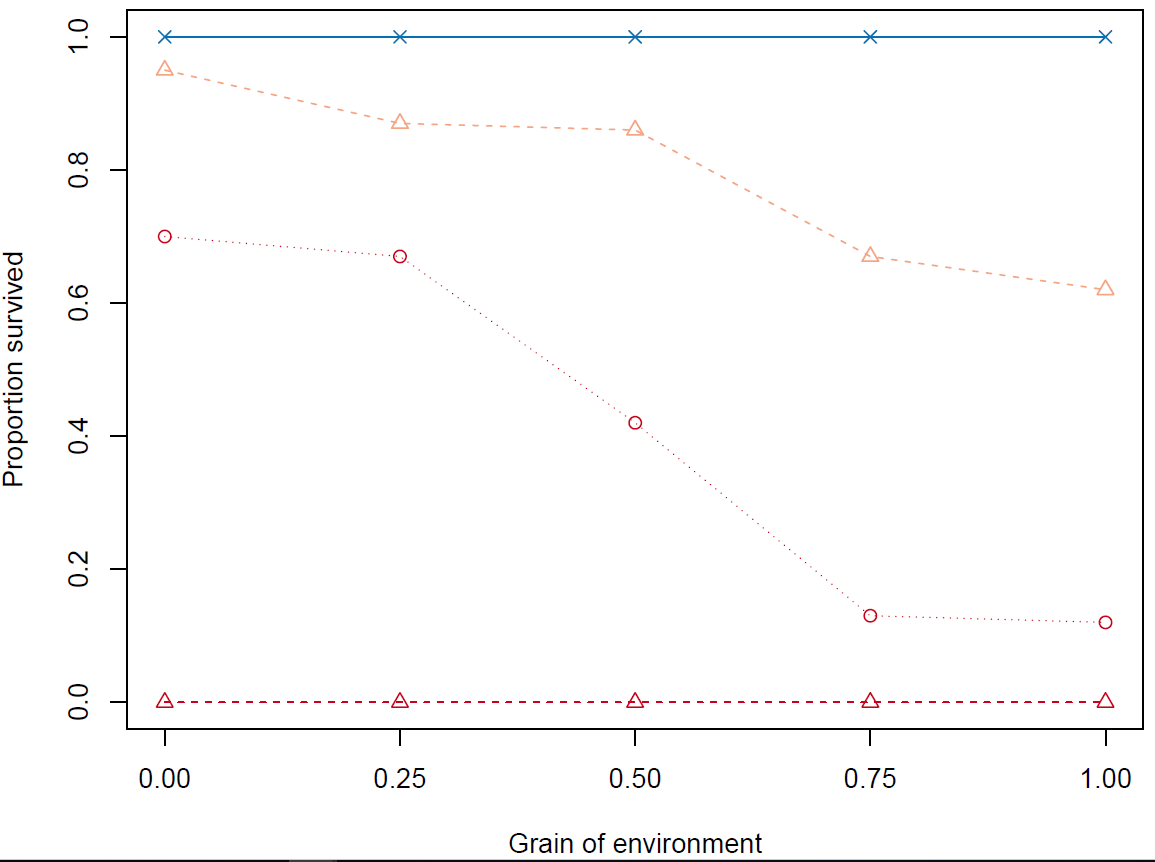


**Figure S2:** Proportion of populations that survived until the end of the simulations in Model 2 with discrete generations, for different environmental grains (*g_e_*). Circles/dotted line: Resource grain *g_r_* = 0.75; triangles/dashed lines: *g_r_* = 1. Colors represent number of decision events per lifetime *n*, red: *n* = 1; orange: *n* = 2. Populations at all other parameter combinations (*n* and *g_r_*) had 100 % survival for all *g_e_*, here represented by blue crosses/solid line. All populations with overlapping generations, and all populations in Model 1 had 100 % survival as well.

## Supplementary table

**Table S1.** List of mathematical notation and parameter values

| Parameter or variable | Description | Range or values |
| --- | --- | --- |
| Model 1 | | |
| *R* | Overall state of resource availability (determining whether the variable strategy gives good or bad payoffs) | {0, 1} |
| *g_r_* | Grain of resources | {0, 0.25, 0.5, 0.75, 1} |
| *r_i_* | Local resource availability for individual *i* | {0 (bad), 1 (good)} |
| *µ* | Mean payoff of the variable strategy | 2 (baseline) |
| *a* | Proportional reduction in payoff for the safe strategy relative to variable strategy | [0, 1), set to 0.1 in figure 2 and 3. |
| *b* | Proportional variability in payoff for the variable strategy | (0, 1], set to 0.9 in figure 2 and 3. |
| Model 2 | | |
| *E* | Overall state of environmental quality (determining energetic state) | [0, 1] |
| *g_e_* | Grain of environment | {0, 0.25, 0.5, 0.75, 1} |
| *x_i_* | Energetic state of individual *i* | [0, 1] |
| Simulation parameters | | |
| *n* | Number of decision events prior to reproduction | {1, 2, 5, 10} |
| *α* | Between-season mortality | Set to 1 or 0.5 in fig. 2 and S1, and 0.5 in fig. 3. |
| *K* | Carrying capacity | 5000 |
| *m_p_* | Mutation rate | Baseline 0.005 |
| *m_σ_* | Mutational size | Baseline 0.05 |

## R code

########################################

## VARIANCE-SENSITIVITY & BET-HEDGING ##

## HAALAND, WRIGHT & RATIKAINEN, 2019 ##

########################################

rm(list=ls())

## Utility function - sigmoid relationship between energetic state and fitness

Utility <- function(x,r) r/(1+exp(-5*(x-0.5))) #x is state; r is upper asymptote.

## sim(): Individual-based simulation.

## Arguments:

## n: number of time steps/instances before reproduction, over which to gather resources.

## alpha: Between-year mortality

## m.rate: Mutation rate. Default 0.005

## m.size: Mutation size. Default 0.05

## K: Carrying capacity. Default 5000. Density dependence is 'ceiling' type.

## T: Number of years/seasons. Default 2000

## reps: Number of replicate simulations. Default 1

## plot: Logical. Whether to plot population trajectories, or not (default not).

## Variant:1 (default): Resources gained relate linearly to fitness.

## 2: Energetic state random, but risky foraging can change it. Sigmoid relation with fitness.

## grain_res: 0: No correlation among patch quality. 1: All patches have same quality. [0,1]

## grain_env: Only in variant 2. Correlation among individual states in a time step. [0,1]

## mu: Average reproductive rate

## a: Penalty of choosing constant patch (only in Variant 1). Default 0.1. (0,1)

## b: Variability of variable patch. Default 0.1. a and b represent proportions of mu. [0,1]

## Title: Plot title. Default blank

sim <- function(n,alpha=0.2,m.rate=0.005,m.size=0.05,K=5000,T=2000,reps=1,plot=FALSE,Variant=1,

grain_res=0,grain_env=0.1,mu=2,b=0.1,a=0.1,title=""){

if(plot){

plot(1:T,rep(1,T),type="n",ylim=c(0,1),ylab="Proportion variance-prone",xlab="Year",main=title)

}

if(Variant==1){

w.s <- c(mu*(1-b),mu*(1+b)) # Payoffs of variable patch, c(bad,good)

w.n <- mu*(1-a) # Payoff of constant patch (non-sensitive)

}else if(Variant==2){

w.s <- c(-0.1,0.1) # Change in state at variable patch, c(bad,good)

w.n <- 0 # Change in state at constant patch

}

nstorage <- zstorage <- matrix(NA,reps,T) # Storage matrices for population sizes and gene values

for(rep in 1:reps){

#Initiate population

N <- K #Starting pop. size is at carrying capacity

pop <- runif(N) #Initiate genes for probability of playing risky strategy.

for(Time in 1:T){

#Record population traits

zstorage[rep,Time] <- mean(as.numeric(pop)) #Record phenotype

nstorage[rep,Time] <- N #Record population size.

strategy <- ifelse(pop>runif(N),TRUE,FALSE) #Strategy played. TRUE=Risky, FALSE=Safe

if(Variant==1){

W <- rep(0,N)

for(Step in 1:n){

this.W <- rep(0,N)

cond <- ifelse(runif(1)<0.5,TRUE,FALSE) #Good (TRUE) or bad (FALSE) resource conditions?

if(grain_res<1){

conds <- sample(c(cond,!cond),size=N,prob=c(0.5+grain_res/2,0.5-grain_res/2),replace=TRUE)

this.W[which(strategy==TRUE & conds==cond)] <- w.s[as.numeric(cond)+1]

this.W[which(strategy==TRUE & conds!=cond)] <- w.s[as.numeric(!cond)+1]

this.W[which(strategy==FALSE)] <- w.n #Payoff for playing safe strategy doesn't depend on cond.

} else{

this.W[which(strategy==TRUE)] <- w.s[as.numeric(cond)+1]

this.W[which(strategy==FALSE)] <- w.n

}

W <- W+this.W

}

}

else if(Variant==2){

W <- rep(0,N)

for(Step in 1:n){

this.W <- rep(0,N)

cond <- runif(1) # Mean environmental condition

lower <- grain_env*cond

upper <- cond + (1-grain_env)*(1-cond)

states <- runif(N,lower,upper) # 'Energetic states' of individuals depend on env.conditions, but can differ more or less depending on grain_env

gamblers <- which(strategy==TRUE & states<0.5) # Only risky individuals in the accel. part of the curve gamble.

env <- ifelse(runif(1)<0.5,1,2) #Resource quality, bad (1) or good (2)

if(grain_res<1){

this.W[gamblers] <- sample(c(w.s[env],w.s[which(w.s!=w.s[env])]),size=length(gamblers),

prob=c(0.5+grain_res/2,0.5-grain_res/2),replace=TRUE)

} else{

this.W[gamblers] <- w.s[env]

}

#Risk averse or non-sensitive have unchanged states.

W <- W+Utility(states+this.W,2*mu) #Fitness payoffs are determined by sigmoid utility function

}

}

if(sum(W)==0){

print(c("Extinct at time",Time))

break

}

#Selection

if(alpha==1){ # If discrete generations (all adults die)

NextN <- ifelse(sum(W)>K,K,round(sum(W)/n))

pop <- sample(pop,size=NextN,prob=W,replace=TRUE)

#Mutation

mut <- which(runif(NextN)<m.rate) #Select mutated individuals

pop[mut] <- rnorm(length(mut),pop[mut],m.size)

}

else{ # If overlapping generations (some adults survive)

Alive <- runif(N)>alpha

Alive <- which(Alive==TRUE) # Alive is a sequence of numbers of the individuals who survived.

#How many 'slots' should be filled (i.e. how many offspring should be produced?)

Offspring <- min(sum(W),K-length(Alive))

#Reproduction. IDs of those who get offspring. If mortality happens before breeding:

IDs <- sample(Alive,size=Offspring,prob=W[Alive],replace=TRUE)

##If mortality happens after breeding (hashtag away if applying):

#IDs <- sample(1:N,size=Offspring,prob=W,replace=TRUE)

#Next generation is a combination of new and old individuals.

pop <- c(pop[IDs],pop[Alive])

#Mutation: Some of the offspring mutate

mut <- which(runif(Offspring)<m.rate) #Select mutated individuals

pop[mut] <- rnorm(length(mut),pop[mut],m.size)

NextN <- length(pop)

}

pop[pop<0] <- 0 # Make sure noone has negative phenotypic values

pop[pop>1] <- 1

#Update population size

N <- NextN

}

print(rep)

if(plot){

lines(1:T,zstorage[rep,],col=rgb(1,0,0,0.1))

}

}

return(list(zstorage,nstorage))

}

#Running the simulation (example)

test <- sim(n=1,alpha=1,reps=20,m.rate=0.1,plot=TRUE,Variant=1,grain_res=0.2,grain_env=1,b=0.9,a=0.1,mu=2)

for(i in 1:20){lines(1:2000,test[[2]][i,1:2000]/5000,col=rgb(0,0,1,0.1))} # Plotting population size

#### Geometric mean calculations for Model 1 ####

# Calculate according to equation 1 and inequality 2

g.mean.calc <- function(mu=1,a=0.1,b=0.1,n=1,type="gm_var"){

store <- numeric(n+1)

for(m in 0:n){

store[m+1] <- ((mu*(1-b))*(n-m)+(mu*(1+b))*m)^dbinom(m,n,0.5)

}

gm_var <- prod(store)

gm_const <- n*mu*(1-a)

return(switch(type,"gm_var"=gm_var, #Geometric mean of variable patch

"gm_const"=gm_const, #Geometric mean of constant patch

"cond_a"=1-(gm_var/(n*mu)), #Condition for a below which the constant patch is better

"cond_b"=gm_const/(n*mu))) #Condition for b above which variable patch is better

}

### Heatmap:

ns <- 1:8

bs <- (1:10)/10

store <- matrix(0,length(ns),length(bs))

for(i in 1:8){

for(j in 1:10){

store[i,j] <- g.mean.calc(mu=2,b=bs[j],n=i,type="cond_a")

}

}

par(mfrow=c(1,2),mar=c(4,4,2,0.1))

breaks <- c(seq(0,0.01,by=0.001),seq(0.02,1,by=0.02))

image(x=ns,y=bs,z=store,breaks=breaks,col=grey.colors(60,start=0.05,end=1),bty="L",

xlab=expression("Number of decision events prior to reproduction, "*italic(n)),

ylab=expression("Magnitude of variation at variable patch, "*italic(b)),xaxt="n",yaxt="n")

axis(side=1,at=1:8)

axis(side=2,at=1:10/10)

labs <- c(0.005,0.01,0.025,0.05,0.1,0.25,0.5)

contour(ns,bs,store,add=TRUE,levels=labs,col="White")

#Scale bar

test <- matrix(60,10,60)

test[1,] <- 1:60

image(x=1:10,y=1:60,z=test,col=grey.colors(60,start=0.05,end=1),xlab="",ylab="",xaxt="n",yaxt="n",bty="n")

labs <- c(0.002,0.02,breaks[21],0.5,0.9)

text(x=rep(3,length(labs)),y=which(breaks %in% labs),labs)
